# CEP55 promotes prostate cancer progression *via* TPX2-dependent activation of AURKA–PI3K–AKT signaling and inhibition of ferroptosis

**DOI:** 10.1016/j.jbc.2026.111218

**Published:** 2026-01-28

**Authors:** Lizhe Xu, Jinzhuo Ning, Jinrun Wang, Pengcheng Jiang, Xu Zhang, Fan Cheng

**Affiliations:** 1 Department of Urology, Renmin Hospital of Wuhan University, Wuhan, Hubei Province, PR China; 2 Department of Urology, Chinese PLA General Hospital, Beijing, PR China

**Keywords:** CEP55, TPX2, AURKA, PI3K–AKT pathway, ferroptosis, prostate cancer

## Abstract

Prostate cancer (PCa) is a highly heterogeneous malignancy with variable clinical outcomes. Centrosomal protein 55 (CEP55) has been involved in the progression of multiple cancers, but its function in PCa is still largely uncharacterized. CEP55 expression was evaluated in clinical samples and cell lines *via* bioinformatics analysis, quantitative RT–PCR, and Western blot. Functional assessments, such as wound healing, Cell Counting Kit-8, and Transwell invasion, were carried out to evaluate the impact of CEP55 upregulation or knockdown on PCa cell growth and metastasis. Coimmunoprecipitation was utilized to observe the interaction between CEP55 and TPX2 microtubule nucleation factor. The role of CEP55 in ferroptosis regulation was assessed by measuring IC_50_ values of ferroptosis inducers, lipid reactive oxygen species levels, and the expression of ferroptosis-correlated proteins, solute carrier family 7 member 11 (SLC7A11) and glutathione peroxidase 4 (GPX4). Xenograft tumor models were created to evaluate the *in vivo* effects of CEP55 suppression. CEP55 showed a significant upregulation in PCa tissues and cell lines. CEP55 overexpression was linked to advanced clinicopathological features and poor prognosis. Mechanistically, CEP55 interacted with TPX2 to activate the aurora kinase A (AURKA)–PI3K–AKT signaling cascade. This activation led to increased expression of SLC7A11 and GPX4, reduced lipid reactive oxygen species accumulation, and conferred resistance to ferroptosis. Pharmacological inhibition of the TPX2–AURKA interaction with CAM2602 reversed these effects. *In vivo* trials illustrated that CEP55 knockdown inhibited tumor growth and downregulated key proteins in the TPX2–AURKA–PI3K–AKT and ferroptosis resistance pathway. Our findings demonstrate that CEP55 enhances PCa progression by stimulating the TPX2–AURKA–PI3K–AKT signaling pathway and inhibiting ferroptosis. Targeting this axis may represent a potential therapeutic approach for PCa.

Prostate cancer (PCa) is one of the most common malignancies in males globally, involved substantially in cancer-associated mortality and morbidity (1). Current early detection primarily relies on prostate-specific antigen (PSA) testing, digital rectal examination, and increasingly, advanced imaging techniques, such as multiparametric MRI (2). Standard treatments for localized disorder comprise active surveillance, radical prostatectomy, and radiotherapy, whereas metastatic PCa is managed with androgen deprivation therapy, chemotherapy, and novel hormonal agents, like abiraterone and enzalutamide (3). Despite these advances, the molecular heterogeneity of PCa—ranging from indolent to highly aggressive forms—poses persistent challenges in prognosis and therapeutic decision making (4). A deeper comprehension of the underlying mechanisms driving tumor initiation, progression, and therapeutic resistance is therefore urgently needed to refine clinical strategies and improve patient outcomes.

Centrosomal protein 55 (CEP55), located on chromosome 10q23.33, is a crucial component of the midbody during cytokinesis and belongs to the centrosome-associated protein family (5). CEP55 is a main regulator of cytokinesis, having a vital role in the final stages of cell division by promoting abscission and ensuring proper chromosomal segregation (6). Beyond its physiological function, CEP55 has been implicated in tumorigenesis across multiple cancer types, where its overexpression is frequently correlated with enhanced proliferation, genomic instability, and poor clinical outcomes (7). In endometrial malignancy, CEP55 enhances cell growth and suppresses apoptosis, partly through suppressing Foxo1 signaling (8). In gallbladder malignancy, CEP55 is determined as a hub gene and is related to advanced stage and poor survival; its knockdown triggers G2/M arrest, DNA damage, and apoptosis *via* modulation of AKT serine/threonine kinase 1 and extracellular signal–regulated kinase pathways (9). In colorectal malignancy, CEP55 contributes to immune exclusion and resistance to immune checkpoint inhibitors, whereas its deletion enhances antitumor immunity and sensitizes tumors to anti-PD1 therapy (10). However, despite its well-known oncogenic functions in other malignancies, the functional significance and molecular mechanisms of CEP55 in PCa are still largely unknown. Elucidating its contribution to PCa pathogenesis could uncover novel therapeutic vulnerabilities in this disease.

This investigation aims to illustrate the oncogenic function of CEP55 in PCa by investigating its aberrant expression patterns, functional mechanisms in tumor progression, and potential clinical applications as a predictive biomarker or a therapeutic target.

## Results

### CEP55 is overexpressed in PCa tissues and cell lines

**Figure 1:**
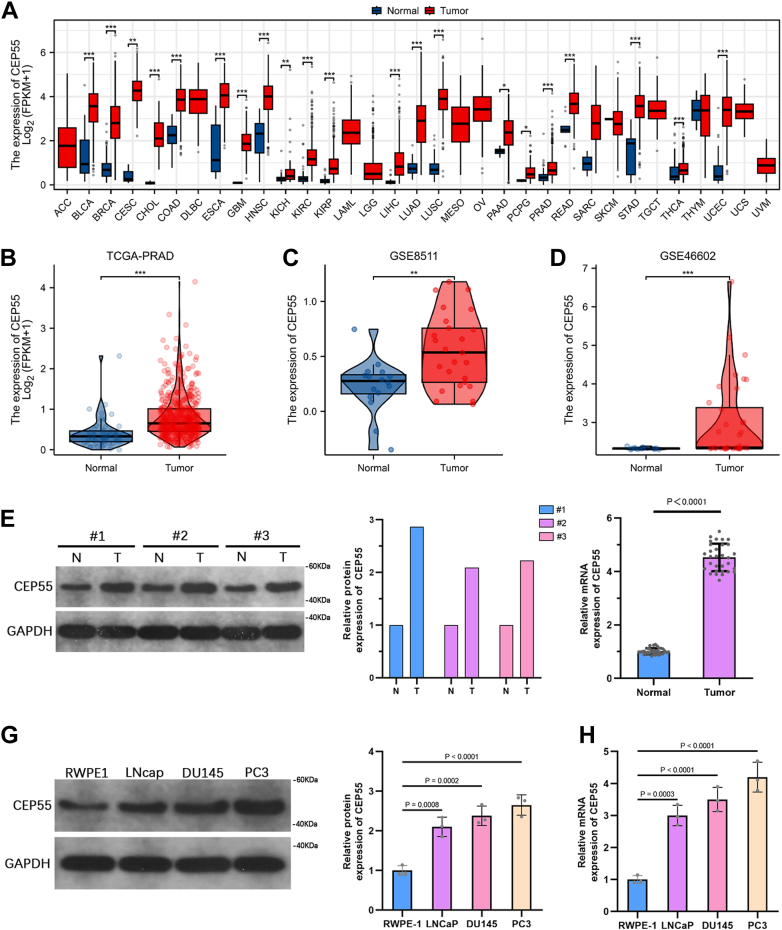
**CEP55 is overexpressed in PCa tissues and cell lines.***A*, pan-cancer analysis of CEP55 expression across TCGA datasets. *B*, comparison of CEP55 expression between tumor and neighboring healthy tissues in the TCGA-PRAD cohort. *C* and *D*, CEP55 concentrations in PCa tissues compared with healthy tissues based on GEO datasets GSE8511 and GSE46602. *E* and *F*, WB and qRT-PCR analyses of CEP55 expression in tumor and neighboring healthy tissues from clinical PCa specimens. *G* and *H*, WB and qRT–PCR analyses of CEP55 expression in RWPE-1 and PCa cell lines (LNCaP, DU145, and PC3). The data are presented as the mean ± SD. Unpaired *t* tests were employed to compare two groups. One-way ANOVA with Tukey's test was used for comparisons involving more than two groups. ∗*p* < 0.05; ∗∗*p* < 0.01; and ∗∗∗*p* < 0.001. CEP55, centrosomal protein 55; GEO, Gene Expression Omnibus; PCa, prostate cancer; PRAD, prostate adenocarcinoma; qRT–PCR, quantitative RT–PCR; TCGA, The Cancer Genome Atlas; WB, Western blot.

Using The Cancer Genome Atlas (TCGA) pan-cancer dataset, we found that CEP55 was significantly higher in various tumor tissues than their corresponding healthy counterparts (Fig. 1*A*). In the TCGA-Prostate Adenocarcinoma (PRAD) cohort, CEP55 expression was markedly higher in prostate tumor tissues than in neighboring nontumor tissues (Fig. 1*B*). Constantly, analysis of independent gene expression datasets from the Gene Expression Omnibus (GEO) database (GSE8511 and GSE46602) validated higher CEP55 levels in PCa samples than healthy controls (Fig. 1, *C* and *D*). To further confirm these outcomes at the protein concentration, we performed Western blot (WB) and quantitative RT–PCR (qRT–PCR) analyses on clinical specimens collected from patients with PCa. Both assays demonstrated that CEP55 expression showed a significant elevation in tumor tissues compared with neighboring healthy tissues (Fig. 1, *E* and *F*). Also, CEP55 expression was evaluated in human prostate epithelial cells (RWPE-1) and PCa cell lines (LNCaP, DU145, and PC3). WB and qRT–PCR outcomes illustrated that CEP55 expression was noticeably increased in malignant cell lines compared with RWPE-1 cells, with the highest levels observed in DU145 and PC3 cells (Fig. 1, *G* and *H*). Thus, DU145 and PC3 cells were selected for the following functional trials.

### CEP55 expression is linked to poor prognosis in PCa

**Figure 2:**
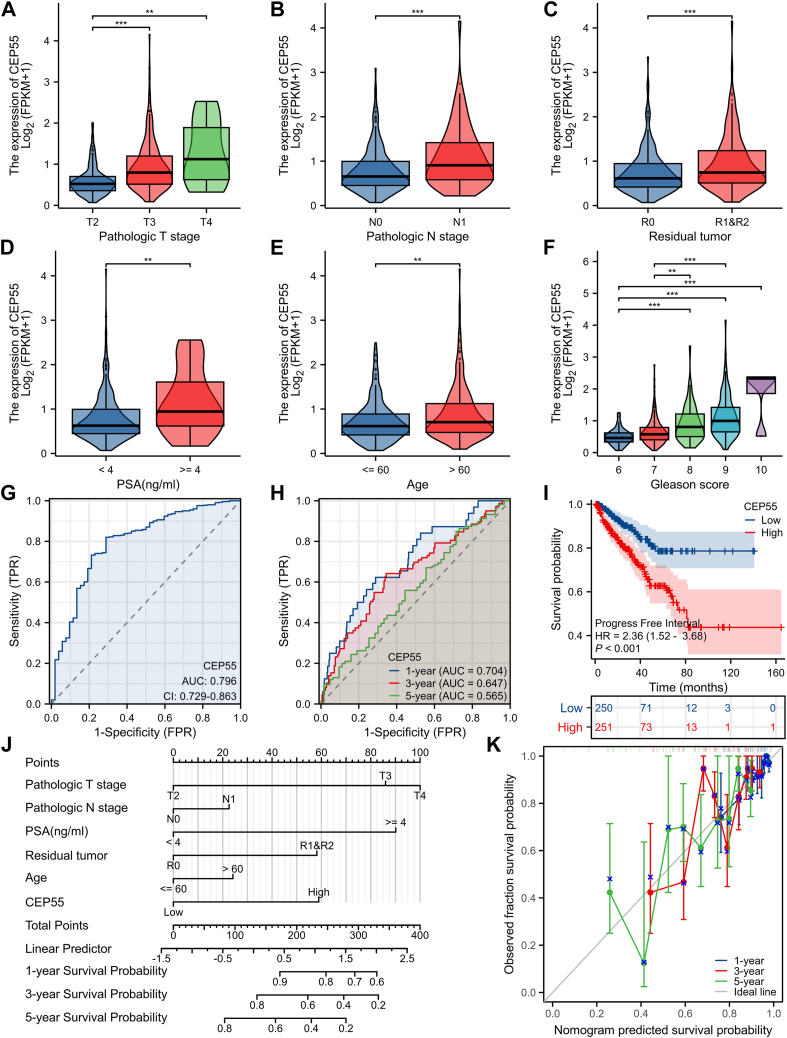
**CEP55 expression is linked to a bad prognosis in PCa.***A*–*F*, expression levels of CEP55 in relation to clinicopathological parameters, including T stage, N stage, residual tumor, PSA level, age, and Gleason score, based on the TCGA-PRAD cohort. *G*, ROC curve analysis of CEP55 for the diagnosis of PCa. *H*, time-dependent ROC analysis evaluating the predictive value of CEP55 for 1-, 3-, and 5-year PFI in PCa individuals. *I*, Kaplan–Meier survival analysis of PFI in individuals with upregulated *versus* downregulated CEP55 expression. *J*, construction of a nomogram integrating CEP55 expression and independent clinical risk factors (T stage, M stage, PSA level, residual tumor, and age) for forecasting 1-, 3-, and 5-year PFI in PCa individuals. *K*, calibration curves assessing the agreement between predicted and observed survival probabilities based on the nomogram model. The data are presented as the mean ± SD. Unpaired *t* tests were employed to compare two groups. ∗*p* < 0.05; ∗∗*p* < 0.01; and ∗∗∗*p* < 0.001. CEP55, centrosomal protein 55; PCa, prostate cancer; PFI, progression-free interval; PRAD, prostate adenocarcinoma; PSA, prostate-specific antigen; ROC, receiver operating characteristic; TCGA, The Cancer Genome Atlas.

TCGA-PRAD cohort analysis revealed a significant relationship between higher CEP55 levels and advanced T stage, N stage, residual tumor, elevated PSA levels, older age, and higher Gleason scores (Fig. 2, *A*–*F*), indicating a potential link between CEP55 and tumor progression. Receiver operating characteristic (ROC) curve analysis demonstrated that CEP55 had a moderate diagnostic value for PCa, with an area under the curve of 0.796 (Fig. 2*G*). Time-dependent ROC analysis further showed that CEP55 expression had predictive value for 1-, 3-, and 5-year survival probabilities, with a stronger performance in short-term prediction compared with long-term prediction (Fig. 2*H*). Kaplan–Meier survival analysis illustrated that individuals with increased CEP55 expression showed a significant reduction in progression-free interval (PFI) compared with those with decreased expression (Fig. 2*I*). To develop a clinically applicable prognostic model, a nomogram incorporating CEP55 expression and independent clinical risk factors (T stage, N stage, PSA level, residual tumor, and age) was constructed to forecast the 1-, 3-, and 5-year PFI probabilities of PCa individuals (Fig. 2*J*). Observed survival probabilities were in good agreement with the nomogram-predicted probability, according to calibration curves (Fig. 2*K*). The nomogram’s concordance index for overall survival was 0.692 (95% confidence interval, 0.659–0.724), suggesting a reliable predictive performance.

### Overexpression of CEP55 enhances the aggressiveness of PCa cells

**Figure 3:**
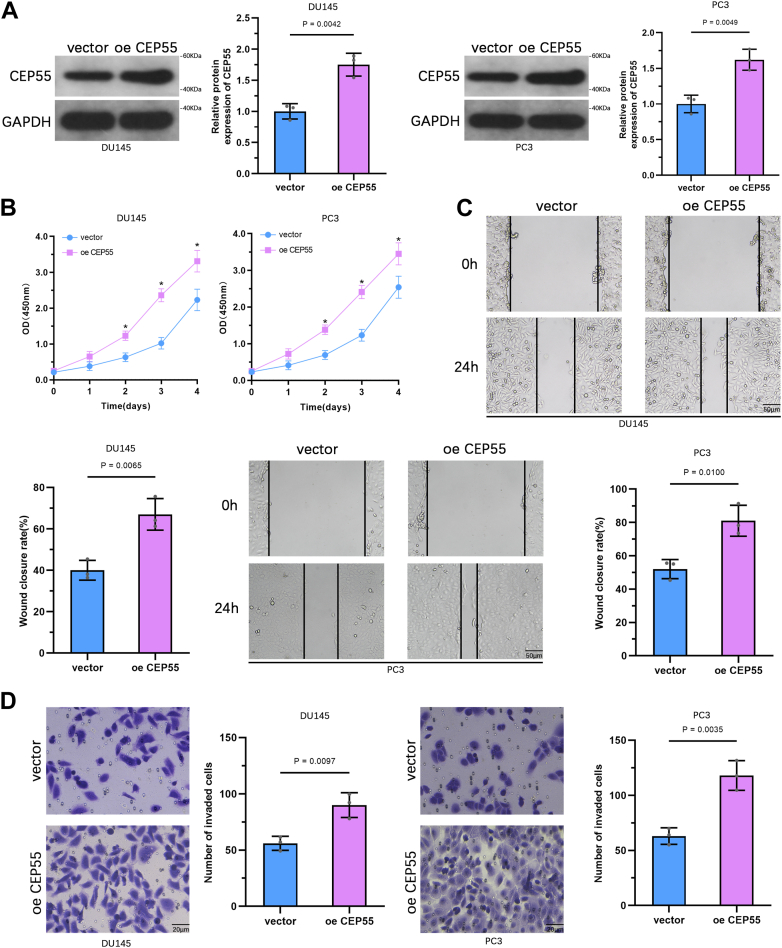
**CEP55 overexpression triggers the aggressiveness of PCa cells.***A*, WB analysis confirming the CEP55 overexpression in DU145 and PC3 cells following lentiviral transduction. *B*, CCK-8 assay detects the proliferative capability of DU145 and PC3 cells after CEP55 overexpression. *C*, wound-healing assay assessing the migratory ability of DU145 and PC3 cells after CEP55 overexpression. *D*, transwell invasion assay assessing the invasive capability of DU145 and PC3 cells after CEP55 overexpression. The data are presented as the mean ± SD. Unpaired *t* tests were employed to compare two groups. ∗*p* < 0.05. CCK-8, Cell Counting Kit-8; CEP55, centrosomal protein 55; PCa, prostate cancer; WB, Western blot.

To examine the functional role of CEP55 in PCa progression, DU145 and PC3 cells were transduced with lentiviral vectors to overexpress CEP55, and the efficiency of overexpression was established by WB analysis (Fig. 3*A*). Cell Counting Kit-8 (CCK-8) assays illustrated that, compared with the control group, CEP55 upregulation significantly improved the proliferative capacity of both DU145 and PC3 cells (Fig. 3*B*). Wound healing assays demonstrated that CEP55-overexpressing cells exhibited faster migration rates than control cells (Fig. 3*C*). Moreover, Transwell assays with Matrigel illustrated that CEP55 overexpression markedly elevated the invasive capability of DU145 and PC3 cells (Fig. 3*D*). These outcomes show that CEP55 has a protumorigenic function in promoting the aggressive behavior of PCa cells *in vitro*.

### Suppression of CEP55 suppresses the aggressiveness of PCa cells

**Figure 4:**
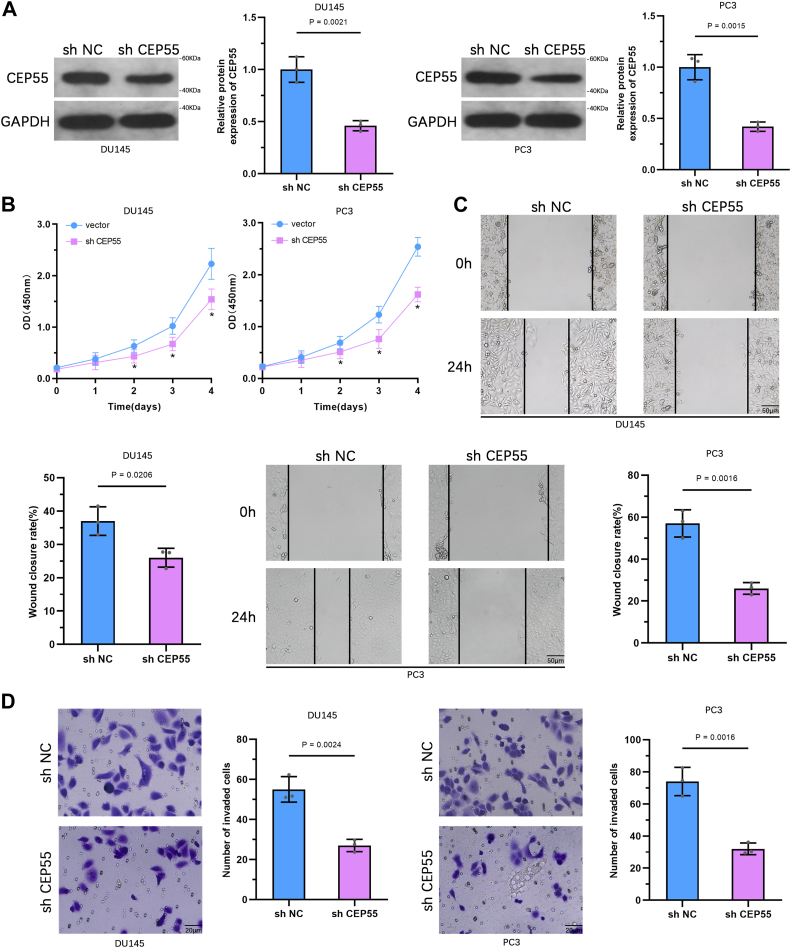
**Suppression of CEP55 suppresses the aggressiveness of PCa cells.***A*, WB analysis validating the suppression of CEP55 in DU145 and PC3 cells after lentiviral transduction. *B*, CCK-8 assay assessing the proliferative capacity of DU145 and PC3 cells after CEP55 silencing. *C*, wound-healing assay evaluating the migratory capability of DU145 and PC3 cells after CEP55 suppression. *D*, transwell invasion assay examining the invasive potential of DU145 and PC3 cells after CEP55 downregulation. The data are presented as the mean ± SD. Unpaired *t* tests were employed to compare two groups. ∗*p* < 0.05. CCK-8, Cell Counting Kit-8; CEP55, centrosomal protein 55; PCa, prostate cancer; WB, Western blot.

To further confirm the CEP55 role in PCa cells, DU145 and PC3 cells were transduced with lentiviral vectors targeting CEP55 to achieve stable knockdown. WB analysis confirmed effective suppression of CEP55 protein expression in both cell lines (Figs. 4*A* and S1*A*). CCK-8 assays showed that CEP55 suppression significantly reduced cell proliferation compared with control cells (Figs. 4*B* and S1*B*). Wound healing assays illustrated a marked reduction in cell migration capacity following CEP55 silencing (Figs. 4*C* and S1*C*). Similarly, Transwell invasion assays illustrated that the invasive capability of DU145 and PC3 cells was significantly impaired after CEP55 knockdown (Figs. 4*D* and S1*D*). Collectively, these outcomes further improve the oncogenic function of CEP55 in promoting PCa cell growth and metastasis.

### Identification and functional enrichment analysis of CEP55 coexpressed genes in PCa

**Figure 5:**
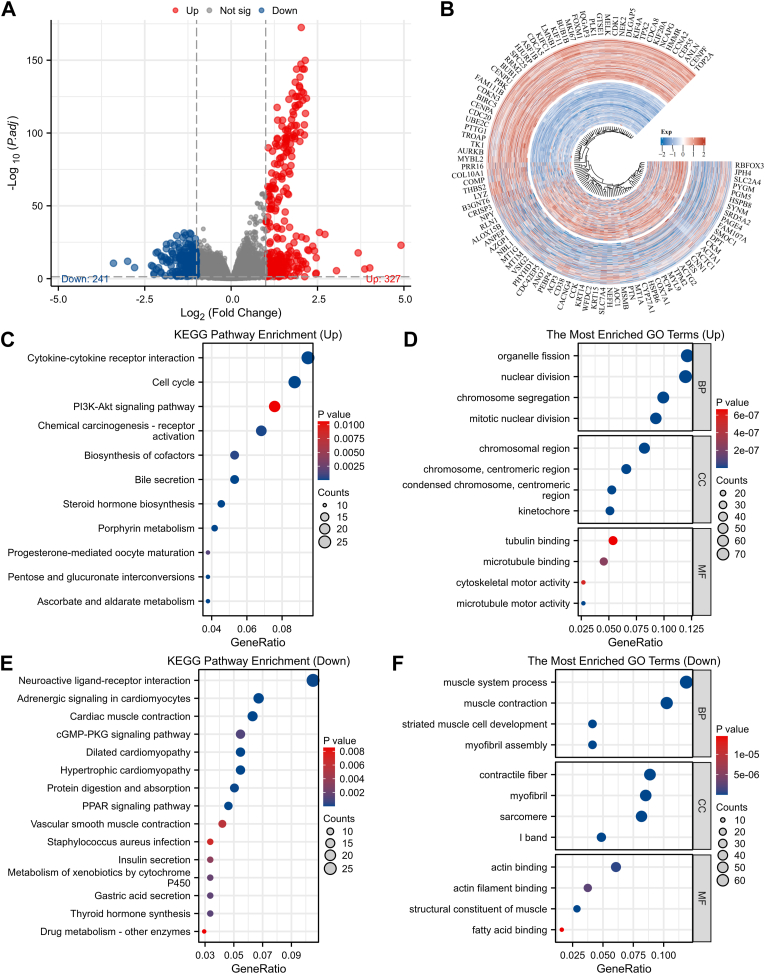
**Identification and functional enrichment analysis of CEP55 coexpressed genes in PCa.***A* and *B*, genes related to CEP55 expression were identified in the TCGA-PRAD dataset using a volcano plot and a heatmap analyses. *C* and *D*, functional enrichment, including KEGG and GO analyses, was performed on genes exhibiting positive correlation with CEP55 in PCa. *E* and *F*, conversely, KEGG and GO enrichment analyses were also carried out on genes showing negative correlation with CEP55 in PCa. CEP55, centrosomal protein 55; GO, Gene Ontology; KEGG, Kyoto Encyclopedia of Genes and Genomes; PCa, prostate cancer; PRAD, prostate adenocarcinoma; TCGA, The Cancer Genome Atlas.

To examine the potential biological functions of CEP55 in PCa, transcriptomic profiles from the TCGA-PRAD dataset were analyzed to identify significantly differentially expressed genes exhibiting strong correlations (both positive and negative) with CEP55 expression (Fig. 5, *A* and *B*). Functional enrichment analysis *via* Gene Ontology and Kyoto Encyclopedia of Genes and Genomes pathways illustrated that the overexpressed genes were primarily enriched in processes, such as organelle fission, PI3K–AKT pathway, and cytokine–cytokine receptor interaction (Fig. 5, *C* and *D*). In contrast, decreased genes were primarily linked to neuroactive ligand–receptor interaction and muscle system process (Fig. 5, *E* and *F*). These outcomes illustrate that CEP55 may participate in PCa progression through the regulation of key cellular processes and signaling pathways involved in tumor proliferation and metastasis.

### CEP55 activates the AURKA–PI3K–AKT pathway in PCa cells

**Figure 6:**
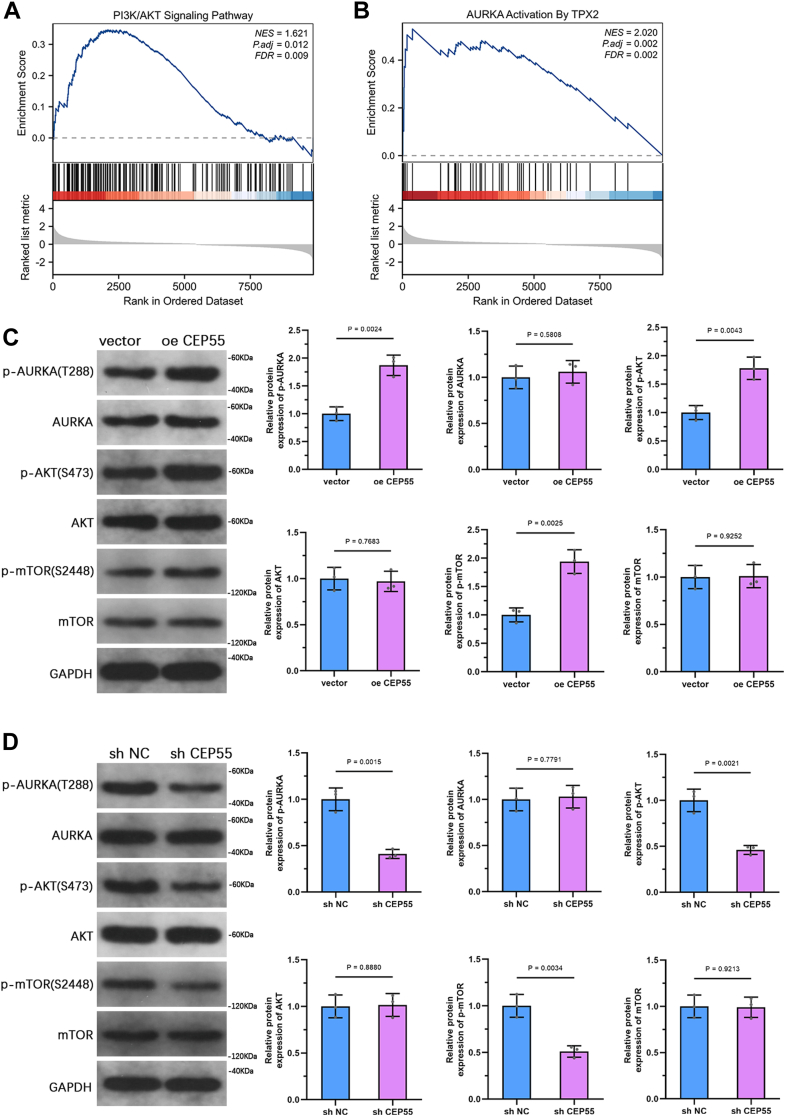
**CEP55 activates the AURK–PI3K–AKT pathway in PCa cells.***A* and *B*, GSEA illustrated that high CEP55 expression showed a significant enrichment in the PI3K–AKT pathway and the TPX2–AURKA axis in the TCGA-PRAD cohort. *C*, WB analysis of phosphorylated and total protein concentrations of AURKA, AKT, and p-mTOR in PC3 cells overexpressing CEP55. *D*, WB analysis of phosphorylated and total protein concentrations of AURKA, AKT, and mTOR in PC3 cells with CEP55 knockdown. The data are presented as the mean ± SD. Unpaired *t* tests were employed to compare two groups. ∗*p* < 0.05. AURKA, aurora kinase A; CEP55, centrosomal protein 55; GSEA, gene set enrichment analysis; PCa, prostate cancer; p-mTOR, phosphorylated mechanistic target of rapamycin; PRAD, prostate adenocarcinoma; TCGA, The Cancer Genome Atlas; WB, Western blot.

Gene set enrichment analysis (GSEA) illustrated that CEP55 overexpression was significantly related to the stimulation of the PI3K–AKT pathway and the TPX2–AURKA axis in PCa (Fig. 6, *A* and *B*). To further confirm this functional relationship, WB analysis was conducted in PC3 cells following CEP55 overexpression, showing increased phospho-AURKA (p-AURKA), phospho-AKT (p-AKT), and phosphorylated mechanistic target of rapamycin (p-mTOR) levels, whereas total AURKA, AKT, and mTOR levels continued to be relatively unaffected (Fig. 6*C*). Conversely, silencing of CEP55 in PC3 cells resulted in decreased phosphorylation levels of AURKA, AKT, and mTOR (Fig. 6*D*). These outcomes illustrate that CEP55 may improve PCa progression by modulating the AURKA–PI3K–AKT pathway.

### CEP55 is coexpressed with TPX2 and interacts with it in PCa

**Figure 7:**
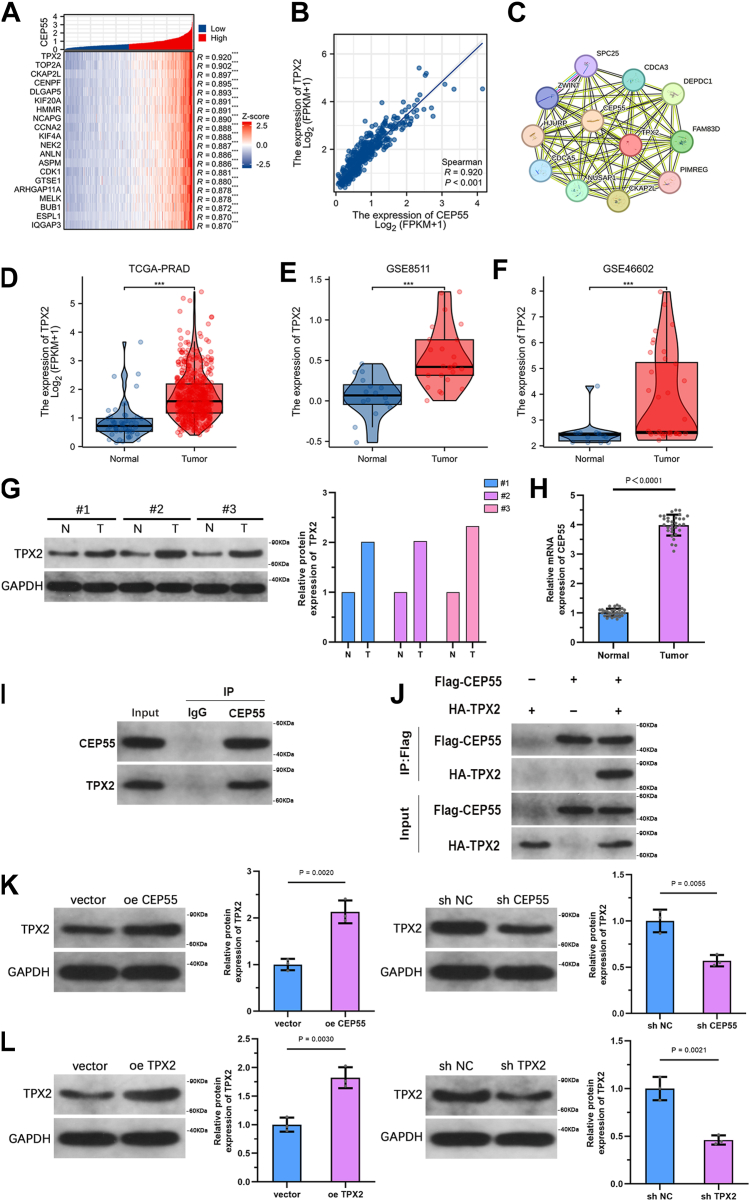
**CEP55 is coexpressed with TPX2 and interacts with it in PCa.***A*, hierarchical clustering and heatmap showing the top 20 genes coexpressed with CEP55 in PCa based on the TCGA-PRAD dataset. *B*, Spearman's correlation analysis of CEP55 and TPX2 levels in PCa tissues. *C*, STRING database analysis was utilized to forecast potential protein–protein interactions between CEP55 and TPX2. *D*, comparison of TPX2 expression between tumor and neighboring healthy tissues using the TCGA-PRAD dataset. *E* and *F*, TPX2 levels in PCa and healthy tissues were analyzed *via* the GEO datasets GSE8511 and GSE46602. *G* and *H*, WB and qRT–PCR were conducted to evaluate TPX2 expression in clinical PCa tissues and matched neighboring healthy tissues. *I* and *J*, Co-IP assays, using both endogenous and exogenous protein extracts, were conducted to assess the interaction between CEP55 and TPX2. *K*, WB analysis was used to detect TPX2 expression following CEP55 overexpression or knockdown in PC3 cells. *L*, WB analysis was conducted to confirm the efficiency of TPX2 overexpression and knockdown in PC3 cells. The data are presented as the mean ± SD. Unpaired *t* tests were employed to compare two groups. ∗*p* < 0.05. CEP55, centrosomal protein 55; Co-IP, coimmunprecipitation; GEO, Gene Expression Omnibus; PCa, prostate cancer; PRAD, prostate adenocarcinoma; qRT–PCR, quantitative RT–PCR; TCGA, The Cancer Genome Atlas; WB, Western blot.

To further explore genes coexpressed with CEP55 in PCa, transcriptomic profiles from the TCGA-PRAD dataset were analyzed, and hierarchical clustering was performed. The top 20 genes most strongly correlated with CEP55 expression were visualized using a heatmap (Fig. 7*A*). Spearman's correlation analysis illustrated a strong positive relationship between CEP55 and TPX2 expression (*R* = 0.920), indicating a potential functional relationship (Fig. 7*B*). STRING database analysis further suggested a possible protein–protein interaction between CEP55 and TPX2 (Fig. 7*C*). Consistently, TPX2 expression showed a significant elevation in tumor tissues compared with neighboring healthy tissues, depending on the TCGA-PRAD dataset (Fig. 7*D*). This upregulation was further validated in two independent GEO datasets, GSE8511 and GSE46602 (Fig. 7, *E* and *F*). WB and qRT–PCR analyses of clinical PCa specimens also confirmed elevated TPX2 protein levels in tumor tissues compared with matched neighboring healthy tissues (Fig. 7, *G* and *H*). To assess the direct interaction between CEP55 and TPX2, coimmunoprecipitation (Co-IP) assays were performed using both endogenous and exogenous protein extracts, which demonstrated a physical association between CEP55 and TPX2 (Fig. 7, *I* and *J*). Moreover, WB analysis showed that overexpression of CEP55 in PC3 cells led to increased TPX2 protein levels, whereas knockdown of CEP55 resulted in decreased TPX2 expression (Fig. 7*K*). The efficacy of TPX2 upregulation and downregulation in PC3 cells was confirmed by WB analysis (Fig. 7*L*). Collectively, these results indicate a close functional and molecular interaction between CEP55 and TPX2 in PCa.

### CEP55 promotes resistance to ferroptosis through the regulation of TPX2 in PCa cells

**Figure 8:**
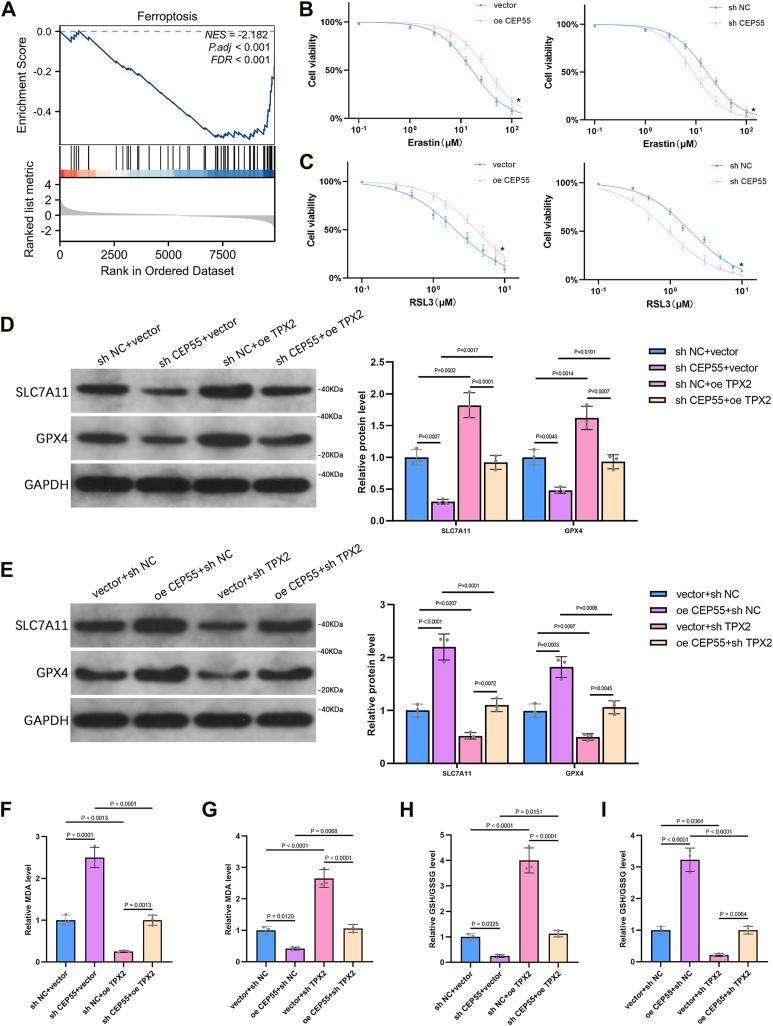
**CEP55 promotes resistance to ferroptosis through the regulation of TPX2 in PCa cells.***A*, GSEA was performed to examine the link between CEP55 expression and the ferroptosis pathway. *B* and *C*, PC3 cells were treated with the ferroptosis inducers, Erastin and RSL3, and the IC_50_ values were determined to assess drug sensitivity following CEP55 overexpression or knockdown. *D*, WB analysis was utilized to observe the level of ferroptosis-associated proteins (SLC7A11 and GPX4) after CEP55 knockdown and TPX2 overexpression. *E*, WB analysis was utilized to detect the expression of SLC7A11 and GPX4 after CEP55 overexpression and TPX2 knockdown. *F*, MDA levels were assessed *via* a commercial assay to evaluate lipid peroxidation in PC3 cells with CEP55 knockdown and TPX2 overexpression. *G*, MDA levels were assessed in PC3 cells with CEP55 overexpression and TPX2 knockdown using the same assay. *H*, the GS–GSSG ratio was measured to assess the cellular redox state in PC3 cells with CEP55 suppression and TPX2 upregulation. *I*, the GSH–GSSG ratio was also evaluated in PC3 cells with CEP55 overexpression and TPX2 knockdown. Unpaired *t* tests were employed to compare two groups. One-way ANOVA with Tukey's test was used for comparisons involving more than two groups. ∗*p* < 0.05. CEP55, centrosomal protein 55; GPX4, glutathione peroxidase 4; GSEA, gene set enrichment analysis; MDA, malondialdehyde; PCa, prostate cancer; WB, Western blot.

GSEA revealed a negative relationship between CEP55 expression and the ferroptosis signaling pathway (Fig. 8*A*). To further assess this relationship, PC3 cells were treated with the ferroptosis inducers, Erastin and RSL3, and the IC_50_ values were determined. Overexpression of CEP55 significantly increased the IC_50_ values of both Erastin and RSL3, indicating reduced sensitivity to ferroptosis induction, whereas CEP55 knockdown had the opposite effect (Fig. 8, *B* and *C*). The expression of ferroptosis-related proteins (SLC7A11 and glutathione peroxidase 4 [GPX4]) was evaluated *via* WB analysis. It was found that CEP55 knockdown led to decreased levels of these proteins, whereas TPX2 overexpression restored their expression (Fig. 8*D*). In contrast, CEP55 overexpression increased SLC7A11 and GPX4 protein levels, and this effect was attenuated by TPX2 knockdown (Fig. 8*E*). To further explore the involvement of TPX2 in ferroptosis regulation, intracellular malondialdehyde (MDA) levels, a marker of lipid peroxidation, were assessed *via* a commercial assay. TPX2 overexpression reversed the increase in MDA levels caused by CEP55 knockdown (Fig. 8*F*), whereas TPX2 silencing significantly increased MDA levels in CEP55-overexpressing PC3 cells (Fig. 8*G*). In addition, the GSH–GSSG ratio was measured to assess the cellular redox state during ferroptosis. TPX2 overexpression restored the declined GSH–GSSG ratio triggered by CEP55 suppression (Fig. 8*H*), whereas TPX2 knockdown attenuated the elevated GSH–GSSG ratio observed in CEP55-overexpressing cells (Fig. 8*I*). These findings suggest that CEP55 may regulate ferroptosis in PCa cells, at least in part, through modulating TPX2 expression and its downstream targets.

### Inhibition of the TPX2–AURKA interaction suppresses CEP55-induced stimulation of the PI3K–AKT pathway and ferroptosis-associated proteins

**Figure 9:**
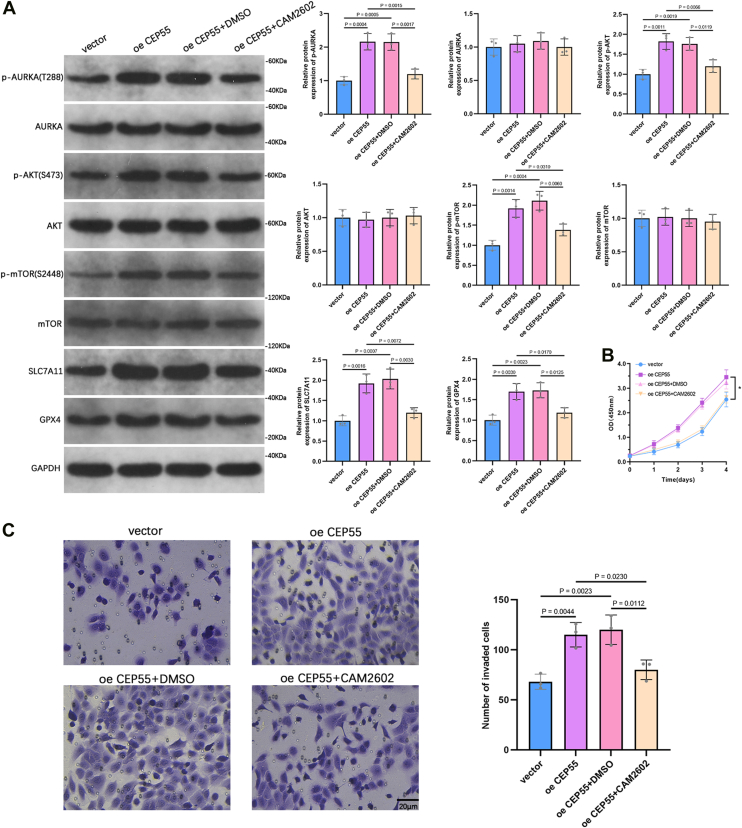
**Inhibition of the TPX2–AURKA interaction suppresses CEP55-induced stimulation of the PI3K–AKT pathway and ferroptosis-associated proteins.***A*, WB analysis was performed to determine the protein levels of AURKA, p-AURKA, AKT, p-AKT, mTOR, p-mTOR, SLC7A11, and GPX4 in PC3 cells overexpressing CEP55 with or without treatment of the AURKA–TPX2 binding inhibitor CAM2602 (20 μM). *B*, CCK-8 assay was conducted to assess cell proliferation in PC3 cells overexpressing CEP55 with or without CAM2602 treatment. *C*, a transwell invasion assay was carried out to determine the invasive capability of PC3 cells overexpressing CEP55 with or without CAM2602 treatment. Unpaired *t* tests were employed to compare two groups. One-way ANOVA with Tukey's test was used for comparisons involving more than two groups. ∗*p* < 0.05. CCK-8, Cell Counting Kit-8; CEP55, centrosomal protein 55; GPX4, glutathione peroxidase 4; mTOR, mechanistic target of rapamycin; p-AURKA, phosphorylated aurora kinase A; WB, Western blot.

To evaluate whether the oncogenic effects of CEP55 are mediated through the TPX2–AURKA interaction, we treated CEP55-overexpressing PC3 cells with CAM2602, a specific inhibitor of this interaction. After a 2-h preincubation with 20 μM CAM2602, WB analysis showed that CEP55 overexpression led to elevated levels of p-AURKA, p-AKT, p-mTOR, SLC7A11, and GPX4, and these enhancements were effectively attenuated by the inhibitor treatment (Fig. 9*A*). We further assessed the functional consequence of this inhibition in a proliferation assay, where CAM2602 was added once at the start and maintained throughout the experiment. The CCK-8 assay confirmed that CAM2602 treatment significantly reduced the enhanced growth ability induced by CEP55 overexpression (Fig. 9*B*). Transwell invasion assays further demonstrated that CAM2602 treatment suppressed the increased invasive capacity observed in CEP55-overexpressing PC3 cells (Fig. 9*C*). These findings indicate that targeting the AURKA–TPX2 interaction can counteract the molecular and phenotypic changes induced by CEP55 overexpression in PCa cells.

### CEP55 suppression inhibits tumor growth *in vivo*

**Figure 10:**
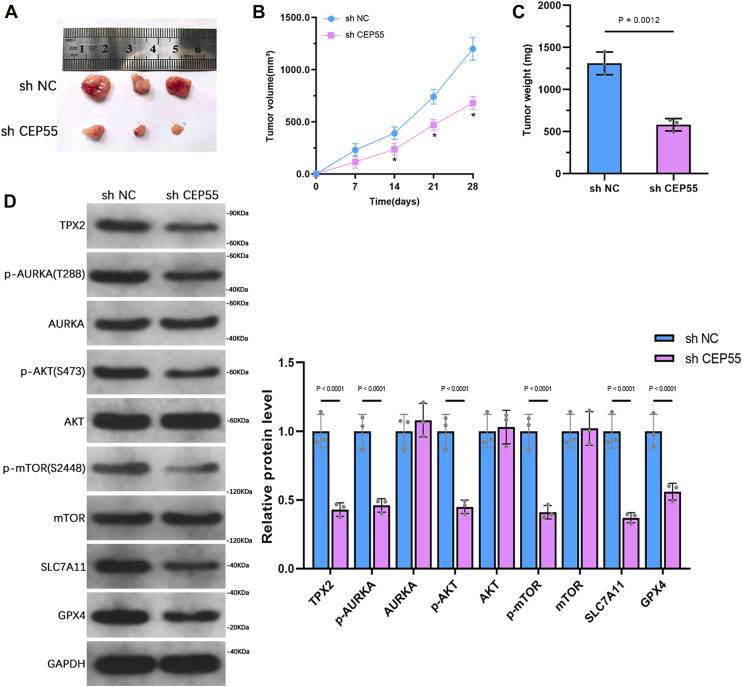
**CEP55 knockdown suppresses tumor growth *in vivo*.***A*, subcutaneous tumor xenograft model was created by injecting PC3 cells with stable CEP55 suppression or control cells into nude mice, followed by tumor collection after 28 days. *B*, tumor growth was monitored by measuring tumor volume at regular intervals during the 28-day period. *C*, tumor weight was observed postexcision at the end of the trial. *D*, WB analysis was performed to observe the concentrations of TPX2, AURKA, p-AURKA, AKT, p-AKT, mTOR, p-mTOR, SLC7A11, and GPX4 in the harvested tumor tissues. The data are presented as the mean ± SD. Unpaired *t* tests were employed to compare two groups. ∗*p* < 0.05. AURKA, aurora kinase A; CEP55, centrosomal protein 55; GPX4, glutathione peroxidase 4; mTOR, mechanistic target of rapamycin; p-AKT, phospho-AKT; p-AURKA, phosphorylated AURKA; p-mTOR, phosphorylated mTOR; WB, Western blot.

To assess the CEP55 role in tumor growth *in vivo*, a subcutaneous injection of PC3 cells with stable CEP55 suppression and control PC3 cells was administered into nude mice. Tumors were harvested after 28 days (Fig. 10*A*). Tumor size was measured at regular intervals, and the outcomes illustrated that, compared with the control group, CEP55 suppression significantly suppressed tumor growth (Fig. 10*B*). After tumor excision, tumor weight was measured, and the data illustrated that CEP55 knockdown resulted in a significant lessening in tumor weight (Fig. 10*C*). WB analysis of the harvested tumor tissues revealed reduced expression of TPX2, p-AURKA, p-AKT, p-mTOR, SLC7A11, and GPX4 in the CEP55 suppression group compared with the control group (Fig. 10*D*). These *in vivo* findings confirm the tumor-promoting role of CEP55 and support its regulation of the TPX2–AURKA axis and ferroptosis resistance pathway within a physiological context.

## Discussion

PCa is a complex and multifaceted disease that predominantly affects older men, often developing slowly over many years (11). Risk factors comprise age, family history, and certain genetic mutations, with symptoms typically emerging in more advanced stages, such as difficulty urinating or bone pain (12). The diversity in disease presentation underscores the importance of identifying the biological processes that govern its development and evolution. Ultimately, unraveling the mechanisms behind PCa is crucial for improving diagnosis, personalizing treatment, and enhancing survival rates.

CEP55 was originally known as an abscission component that regulates cellular segregation during cytokinesis (13). CEP55 is a crucial protein involved in the process of cell division, where it interacts with components of the endosomal sorting complex required for transport family to facilitate the contraction of the midbody during cytokinesis, ensuring proper separation of daughter cells (14). Accumulating evidence suggests that CEP55 has a significant function in midbody-dependent cellular functions, including centrosome replication and the regulation of cell cycle progression (15). Moreover, CEP55 has been shown to contribute to the autophagic degradation of midbody remnants, a process that is closely linked to the enhancement of cancer cell pluripotency and stemness (16, 17). CEP55 exhibits oncogenic roles in multiple tumor types. In hepatocellular carcinoma, CEP55 is overexpressed and correlates with poor prognosis, tumor progression, and immune infiltration (macrophages, B cells, and CD4+/CD8+ T cells), with its expression potentially regulated by DNA hypomethylation and linked to pathways like cell cycle, mTOR, and vascular endothelial growth factor signaling (18). In non–small cell lung malignancy, elevated CEP55 expression predicts adverse survival outcomes and synergizes with AP3B to serve as a novel prognostic biomarker (19). For anaplastic thyroid cancer, CEP55 is highly expressed, promotes tumor progression, and its downregulation inhibits PI3K–AKT phosphorylation, identifying it as an independent prognostic indicator and potential therapeutic target (20). Our results illustrated that CEP55 showed a significant upregulation in PCa tissues and cell lines, and its expression is strictly linked to advanced clinicopathological features and poor prognosis. Functional studies *in vitro* revealed that CEP55 triggers cell growth, migration, and invasion, whereas its knockdown inhibits these malignant behaviors. These outcomes indicate that CEP55 has an oncogenic function in PCa progression.

We discovered through comprehensive bioinformatics analysis that overexpression of CEP55 is linked to the PI3K–AKT pathway and the TPX2–AURKA axis. The PI3K–AKT pathway is a central regulator of key cellular functions, such as survival, metabolism, and growth (21). This pathway is typically stimulated by extracellular stimuli, including growth factors and hormones, resulting in a cascade of intracellular phosphorylation events initiated by PI3K and mediated by its downstream effector AKT (22). Under normal conditions, the PI3K–AKT pathway maintains cellular homeostasis and responds to environmental cues with precise control. However, its dysregulation is frequently associated with numerous human malignancies, including breast (23), gastric (24), colorectal (25), lung cancers (26), and PCa (27), contributing to unchecked cell proliferation and resistance to apoptosis. The TPX2–AURKA axis has a vital function in regulating mitosis and is frequently implicated in various types of cancer (28). TPX2, a microtubule-associated protein, exhibits cell cycle–dependent expression, with levels rising during the G2 phase and peaking in mitosis, before rapidly declining as cells exit mitosis through anaphase-promoting complex/cyclosome– and proteasome-mediated degradation (29). As the most well-characterized activator of AURKA, TPX2 forms a functional complex that is essential for multiple mitotic processes, such as chromosome alignment, centrosome maturation, spindle assembly, and segregation (30). While AURKA primarily localizes to centrosomes and spindle poles, its activation by TPX2 enhances its ability to regulate early mitotic events (31). Notably, this axis has a significant function in the pathogenesis and progression of numerous malignancies, such as colon cancer (32), esophageal squamous cell carcinoma (33), and PCa (34), which is a promising target for further mechanistic and therapeutic investigations. In bladder malignancy, tumor-derived extracellular vesicles deliver active cathepsin B to endothelial cells, triggering TPX2-mediated phosphorylation of AURKA. This activates PI3K–AKT, upregulates vascular endothelial growth factor A, and drives angiogenesis. Inhibiting TPX2 or AURKA blocks this cascade and its proangiogenic effects (35). Our findings indicate that CEP55 promotes oncogenesis, at least in part, through direct stimulation of the TPX2–AURKA–PI3K–AKT signaling axis. We observed that CEP55 interacts with TPX2, and this interaction enhances AURKA phosphorylation, which in turn activates downstream AKT and mTOR. Importantly, pharmacological inhibition of the TPX2–AURKA interaction using CAM2602 effectively suppressed the stimulation of the PI3K–AKT pathway and reversed the malignant phenotypes triggered by CEP55 overexpression. These data suggest that CEP55 promotes tumor progression *via* modulating the TPX2–AURKA–PI3K–AKT signaling cascade.

Ferroptosis is a form of regulated cell death resulting from iron-dependent lipid peroxidation and distinguished by the ROS accumulation and subsequent damage to cellular membranes (36). It differs morphologically and mechanistically from other forms of cell death, including necrosis and apoptosis. A key feature of ferroptosis is the failure of antioxidant defense systems, particularly those involving GSH, which is essential for the function of GPX4, a central enzyme that reduces lipid peroxides to prevent membrane damage (37). The system xc−, composed of the subunit SLC7A11, is responsible for cystine uptake, which is critical for GSH synthesis. Inhibition of SLC7A11 or depletion of GSH leads to lipid ROS accumulation and the eventual activation of ferroptosis. MDA, a byproduct of lipid peroxidation, serves as a marker of oxidative damage during this process. When GPX4 activity is compromised, either through genetic or pharmacological means, the cell is unable to efficiently convert GSH to its oxidized form, GSSG, further contributing to the buildup of toxic lipid peroxides (38). Ferroptosis has emerged as an important mechanism in cancer biology and holds potential for innovative therapeutic approaches aimed at inducing cell death in tumor cells resistant to conventional treatments (39). Our outcomes indicate that CEP55 participates in regulating ferroptosis in PCa cells. GSEA analysis illustrated a negative relationship between CEP55 expression and ferroptosis-related gene signatures. Functional experiments showed that CEP55 overexpression increased the IC_50_ values of ferroptosis inducers, Erastin and RSL3, indicating a protective effect against ferroptosis, whereas CEP55 knockdown enhanced drug sensitivity. Mechanistically, CEP55 modulated the expression of key ferroptosis regulators, SLC7A11 and GPX4, and these effects were mediated through the TPX2–AURKA–PI3K–AKT signaling pathway. Inhibition of TPX2–AURKA interaction with CAM2602 reversed the changes in ferroptosis-related proteins induced by CEP55 overexpression. Furthermore, alterations in MDA, GSH, and GSSG levels upon CEP55 and TPX2 modulation confirmed the involvement of oxidative stress in this process. These findings indicate that CEP55 contributes to ferroptosis resistance, potentially through the stimulation of the TPX2–AURKA–PI3K–AKT axis.

## Conclusion

Briefly, our investigation demonstrates that CEP55 is elevated in PCa and triggers tumor cell growth and invasion while suppressing ferroptosis through the TPX2–AURKA–PI3K–AKT signaling pathway. Targeting the TPX2–AURKA interaction effectively reverses CEP55-induced oncogenic and ferroptosis-resistant phenotypes, highlighting the potential of CEP55 as a prognostic biomarker and therapeutic target in PCa.

## Experimental procedures

### Bioinformatic analysis

We used TCGA (https://portal.gdc.cancer.gov/) to get RNA sequencing gene expression data and related clinical data. In addition, datasets GSE8511 and GSE46602 from the GEO (https://www.ncbi.nlm.nih.gov/geo/) database were downloaded and evaluated to determine gene expression profiles. The relationship between CEP55 expression and clinicopathological parameters, such as tumor stage (T stage), lymph node stage (N stage), residual tumor, PSA level, age, and Gleason score, was evaluated using the “stats” and “car” packages in R. Diagnostic performance was evaluated by generating ROC curves *via* the “pROC” package. Kaplan–Meier survival analysis was carried out with the “survival” package to evaluate the prognostic value of CEP55. A nomogram incorporating CEP55 expression and significant clinical variables was created *via* the “rms” package to forecast PFI; calibration curves were generated to assess its accuracy, with a 45° line indicating optimal prediction. The nomogram’s discriminative capability was estimated by the concordance index. Functional enrichment analyses, including Gene Ontology, Kyoto Encyclopedia of Genes and Genomes pathways, and GSEA, were conducted using the “clusterProfiler” package. The protein–protein interaction network of CEP55 was inferred *via* the STRING database (https://string-db.org/). All bioinformatics analyses were conducted *via* R, version 4.2.1.

### Clinical samples

Thirty-five paired tumor and neighboring healthy tissue samples were collected from PCa individuals who had radical prostatectomy at the Renmin Hospital of Wuhan University (January 2023–December 2024). All samples were histopathologically confirmed, and each patient provided informed consent before surgery. The study was conducted in accordance with the principles of the Declaration of Helsinki. The Ethics Committee of Renmin Hospital of Wuhan University gave its approval to this research.

### Cell lines and cell culture

The American Type Culture Collection provided the human prostate malignant cell lines, LNCaP, DU145, and PC3, in addition to the normal prostate epithelial cell line, RWPE-1. Keratinocyte-serum-free medium from Gibco was used for RWPE-1 cell culture, whereas RPMI1640 medium from the same company was used for LNCaP, DU145, and PC3 cell maintenance. All media were supplemented with 10% fetal bovine serum (ExCell Bio) and 1% penicillin–streptomycin (HyClone) prior to use. Cells were kept at 37 °C in a humidified condition with 5% CO_2_.

### Cell transfection and lentiviral infection

Lentiviral vectors for stable overexpression or knockdown of CEP55 and TPX2 were brought from GeneChem Co, Ltd. The vectors included CEP55 overexpression (oe CEP55), CEP55 knockdown (sh CEP55 and sh CEP55 #2), TPX2 overexpression (oe TPX2), and TPX2 knockdown (sh TPX2). The target sequences of the shRNAs were as follows: sh CEP55, GCAGCATCAATTGCTTGTAAT; sh CEP55 #2, GCAGCGGGAAGTCTATGTAAA; and sh TPX2, CTAATCTTCAGCAAGCTATTG. Cell infection with the corresponding lentivirus was conducted as per the manufacturer's guidelines, and stable cell lines were selected *via* puromycin. Transfection efficacy was established by qRT–PCR and WB analysis.

### Chemicals and pharmacological inhibition

The ferroptosis inducers, erastin (HY-15763), RSL3 (HY-100218A), and the TPX2–AURKA interaction inhibitor, CAM2602 (HY-168022), were all acquired from MedChemExpress.

For inhibition experiments, a 20 mM stock solution of CAM2602 was prepared in dimethyl sulfoxide, aliquoted, and stored at −80 °C. Following the manufacturer's stability specifications, the stock solution was used within 6 months. Immediately prior to each experiment, the stock was diluted in fresh cell culture medium to achieve the final working concentration of 20 μM.

### WB analysis

Protease and phosphatase inhibitors were applied to the radioimmunoprecipitation assay (RIPA) lysis buffer before protein samples were extracted. Equal amounts of total protein were separated by SDS-PAGE and subsequently transferred to polyvinylidene difluoride membranes. After a 1-h blocking step at room temperature with 5% nonfat milk in Tris-buffered saline with Tween-20 buffer, an overnight incubation of the membranes was conducted with primary antibodies at 4 °C. The subsequent primary antibodies were utilized: CEP55 (1:2000 dilution, ab162993, Abcam), TPX2 (1:1000 dilution, ab252944, Abcam), AURKA (1:1000 dilution, ab108353, Abcam), p-AURKA (Thr288, 1:1000 dilution, 44-1210G, Thermo Fisher Scientific), AKT (1:2000 dilution, ab8933, Abcam), p-AKT (Ser473, 1:2000 dilution, ab81283, Abcam), mTOR (1:1000 dilution, AF6308, Affinity), p-mTOR (Ser2448, 1:1000 dilution, #2971, Cell Signaling Technology), SLC7A11 (1:2000 dilution, ab307601, Abcam), GPX4 (1:2000 dilution, ab125066, Abcam), and GAPDH (1:2000 dilution, ab9485, Abcam). A 1-h incubation of secondary antibodies with horseradish peroxidase (1:5000 dilution, ab6721, Abcam) was conducted with membranes at room temperature after washing. The ChemiDoc Imaging System (Bio-Rad) was utilized to acquire images, and an enhanced chemiluminescence detection system (Thermo Fisher Scientific) was utilized for the visualization of protein bands.

### Quantitative real-time PCR

The TRIzol reagent (Invitrogen) was utilized to extract total RNA from both cultured cells and clinical tissue samples, as per the manufacturer's guidelines. Use of a NanoDrop spectrophotometer allowed for the evaluation of RNA concentration and purity. We used the PrimeScript RT Reagent Kit with genomic DNA Eraser (Takara) to synthesize complementary DNA from 1 μg of total RNA. Applied Biosystems' SYBR Green PCR Master Mix was utilized in a qRT–PCR assay on an Applied Biosystems StepOnePlus Real-Time PCR System. The 2^−ΔΔCt^ technique was utilized to calculate the relative mRNA expression levels, which were normalized to GAPDH. Table 1 illustrates the primer sequences used.

**Table 1:** Primer sequences for qRT–PCR

Gene	Forward (5′-3′)	Reverse (5′-3′)
CEP55	AGTGGGGATCGAAGCCTAGT	TCTCAGCCTCAAGGACTCGA
TPX2	GACTTCCACTTCCGCACAGA	TTAGTCACTCGGGCAGGAGA
GAPDH	GTGGACCTGACCTGCCGTCTAG	GAGTGGGTGTCGCTGTTGAAGTC

### Cell viability assay

As per the guidelines of the manufacturer, the CCK-8 from Dojindo was utilized to assess cell viability. Three thousand cells/well were plated into 96-well plates and allowed to proliferate for 0, 24, 48, 72, and 96 h. An incubation of the plates was conducted at 37 °C for an extra 2 h after 10 μl of CCK-8 solution was applied to every well at each time point. An instrument developed by BioTek was employed to test the absorbance at 450 nm.

### Wound healing assay

Cell migration capacity was evaluated *via* a scratch wound healing assay. Cells were introduced to 6-well plates and cultivated till reaching 90% to 100% confluence. A uniform scratch was formed in the cell monolayer *via* a sterile 10 μl pipette tip. The cells were then rinsed with PBS to eliminate debris and cultivated in fresh medium. Images were taken at 0 and 24 h postscratch *via* an inverted microscope (Olympus). The wound healing rate was determined by assessing the change in scratch width over time *via* ImageJ (National Institutes of Health) software.

### Transwell assay

To evaluate cell migration and invasion, researchers utilized Transwell chambers from Corning (a pore size of 8 μm). In order to conduct migration tests, 2 × 10^4^ cells were introduced into the top chamber using serum-free medium, whereas the bottom chamber was loaded with medium that included 10% fetal bovine serum to act as a chemoattractant. To conduct invasion experiments, the same cell solution was transferred to a top chamber that had already been covered with Matrigel (Corning). Any cells from the top surface of the membrane were eliminated after incubation for 24 or 48 h at 37 °C. The fixation of cells that invaded or migrated to the lower surface was conducted *via* 4% paraformaldehyde, staining was conducted *via* 0.1% crystal violet, and an inverted microscope was utilized to count them (Olympus).

### Co-IP analysis

For endogenous Co-IP, cell lysis was carried out in RIPA buffer enriched with protease inhibitors, and total protein extracts (Input) were subjected to IP with anti-CEP55 antibody or control IgG overnight at 4 °C. Protein A/G Sepharose beads were added to capture immune complexes, followed by WB analysis with anti-TPX2 antibody. For exogenous Co-IP, transient transfection of human embryonic kidney 293T cells was conducted with FLAG-CEP55 and/or HA-TPX2 expression plasmids. After 48 h of transfection, IP of cell lysates was conducted with anti-FLAG antibody, and coprecipitated proteins were observed *via* WB using anti-HA antibody. In both assays, input lysates and IgG controls were included to validate protein expression and exclude nonspecific binding. Resolving immune complexes was conducted by SDS-PAGE, followed by transfer to polyvinylidene difluoride membranes for probing with specific antibodies, with signals visualized using enhanced chemiluminescence.

### Assessment of MDA levels

In order to measure lipid peroxidation, we followed the guidelines provided on the Lipid Peroxidation MDA Assay Kit (Beyotime) and measured MDA levels. Cell collection and lysis were conducted in RIPA buffer; the BCA Protein Assay Kit was employed to establish protein levels. Cooling and centrifugation followed an incubation of the lysate-containing reaction mixture with thiobarbituric acid at 95 °C for 30 min. At 532 nm, a microplate reader (BioTek) was utilized to assess the supernatant's absorbance. The ratio of MDA content to protein concentration was subsequently calculated.

### Measurement of GSH–GSSG levels

The reduced (GSH) and oxidized (GSSG) glutathione concentrations were observed *via* the GSH–GSSG Assay Kit (Beyotime) as per the manufacturer's guidelines. Cell collection and lysis were conducted in the provided assay buffer. The lysates were then deproteinized and centrifuged to obtain clear supernatants. A colorimetric reaction was performed, and the absorbance was assessed at 412 nm *via* a microplate reader (BioTek). The GSH and GSSG levels were assessed depending on the standard curve.

### Xenograft tumor formation assay

Using male BALB/c nude mice that were 6 weeks old, we created a xenograft tumor model to assess the tumorigenic potential of CEP55 knockdown in PC3 cells *in vivo*. A subcutaneous injection of 5 × 10^6^ stably transfected PC3 cells was mixed with 100 μl of PBS and then administered into the flanks of the mice. The cells were either sh NC (a negative control) or sh CEP55. Six mice were found in every group. Every 7 days, the tumor's volume was assessed by measuring its length and width using calipers. Once the 28-day period had passed, the tumors were removed and measured after the mice were sacrificed. Tumor samples were prepared for subsequent study by either fixing them in 4% paraformaldehyde or freezing them in liquid nitrogen. This study followed all protocols and was approved by the Renmin Hospital of Wuhan University's Institutional Animal Care and Use Committee.

### Statistical analysis

GraphPad Prism (GraphPad Software), version 9.0, was utilized to analyze all data. Outcomes are expressed as mean ± SD. Comparisons between groups were conducted *via* Student’s *t* test or one-way ANOVA, according to the number of groups and the experimental design. A *p* value below 0.05 was deemed significant. A minimum of three separate repetitions of each trial were conducted.

## Data availability

The data used and analyzed during the current study are available from the corresponding author on reasonable request.

## Supporting information

This article contains supporting information.

## Ethics statement

All procedures were performed in accordance with protocols approved by the Clinical Research Ethics Committee of Renmin Hospital of Wuhan University (approval no.: WDRY2021-KS010) and relevant ethical guidelines. Animal experiments were approved by the same institution’s Ethical Committee (protocol no.: WDRM 20250301F).

## Conflict of interest

The authors declare that they have no conflicts of interest with the contents of this article.
