# Supplementary material for: CEP55 promotes prostate cancer progression via TPX2-dependent activation of AURKA–PI3K–AKT signaling and inhibition of ferroptosis
Source: J Biol Chem. 2026 Jan 28;302(3):111218. doi: 10.1016/j.jbc.2026.111218 (PMC12925562; doi:10.1016/j.jbc.2026.111218)
Supplement: Supporting information [file mmc1.docx]

**Figure S1. Suppression of CEP55 with a second independent shRNA suppresses the aggressiveness of PCa cells**(A) WB analysis validating the suppression of CEP55 by sh CEP55#2 in DU145 and PC3 cells after lentiviral transduction. (B) CCK-8 assay assessing the proliferative capacity of DU145 and PC3 cells after CEP55 silencing with sh CEP55#2. (C) Wound-healing assay evaluating the migratory capability of DU145 and PC3 cells after CEP55 suppression with sh CEP55#2. (D) Transwell invasion assay examining the invasive potential of DU145 and PC3 cells after CEP55 downregulation with sh CEP55#2. The data are presented as the mean ± SD. Unpaired t-tests were employed to compare two groups.* p < 0.05.

**Figure S2. CEP55 promotes resistance to ferroptosis through the regulation of TPX2 in DU145 cells**

(A) WB analysis was performed to assess the expression levels of ferroptosis-associated proteins (SLC7A11 and GPX4) in DU145 cells following CEP55 knockdown and TPX2 overexpression. (B) WB analysis was conducted to evaluate the expression levels of SLC7A11 and GPX4 in DU145 cells following CEP55 overexpression and TPX2 knockdown. Unpaired t-tests were employed to compare two groups. One-way ANOVAs with Tukey test was used for comparisons involving more than two groups. * p < 0.05.
